# Heat Stress Dictates Microbial Lipid Composition along a Thermal Gradient in Marine Sediments

**DOI:** 10.3389/fmicb.2017.01550

**Published:** 2017-08-22

**Authors:** Miriam Sollich, Marcos Y. Yoshinaga, Stefan Häusler, Roy E. Price, Kai-Uwe Hinrichs, Solveig I. Bühring

**Affiliations:** ^1^University of Bremen, MARUM Center for Marine Environmental Sciences Bremen, Germany; ^2^Institute of Chemistry, University of São Paulo São Paulo, Brazil; ^3^Department of Molecular Ecology, Max Planck Institute for Marine Microbiology Bremen, Germany; ^4^School of Marine and Atmospheric Sciences, Stony Brook University, Stony Brook NY, United States

**Keywords:** membrane lipids, heat stress, bioenergetics, bacteria, archaea, shallow-water hydrothermal sediments, membrane fluidity/permeability, adaptation

## Abstract

Temperature exerts a first-order control on microbial populations, which constantly adjust the fluidity and permeability of their cell membrane lipids to minimize loss of energy by ion diffusion across the membrane. Analytical advances in liquid chromatography coupled to mass spectrometry have allowed the detection of a stunning diversity of bacterial and archaeal lipids in extreme environments such as hot springs, hydrothermal vents and deep subsurface marine sediments. Here, we investigated a thermal gradient from 18 to 101°C across a marine sediment field and tested the hypothesis that cell membrane lipids provide a major biochemical basis for the bioenergetics of archaea and bacteria under heat stress. This paper features a detailed lipidomics approach with the focus on membrane lipid structure-function. Membrane lipids analyzed here include polar lipids of bacteria and polar and core lipids of archaea. Reflecting the low permeability of their ether-linked isoprenoids, we found that archaeal polar lipids generally dominate over bacterial lipids in deep layers of the sediments influenced by hydrothermal fluids. A close examination of archaeal and bacterial lipids revealed a membrane quandary: not only low permeability, but also increased fluidity of membranes are required as a unified property of microbial membranes for energy conservation under heat stress. For instance, bacterial fatty acids were composed of longer chain lengths in concert with higher degree of unsaturation while archaea modified their tetraethers by incorporation of additional methyl groups at elevated sediment temperatures. It is possible that these configurations toward a more fluidized membrane at elevated temperatures are counterbalanced by the high abundance of archaeal glycolipids and bacterial sphingolipids, which could reduce membrane permeability through strong intermolecular hydrogen bonding. Our results provide a new angle for interpreting membrane lipid structure-function enabling archaea and bacteria to survive and grow in hydrothermal systems.

## Introduction

Temperature exerts a first-order control on microbial populations, as cell physiology and biochemistry are adapted to specific temperature ranges (Rothschild and Mancinelli, 2001; Schrenk et al., 2008). For instance, archaea and bacteria that use protons and/or sodium as coupling ions for bioenergetics may be affected under elevated temperatures since the ion permeability of biological membranes increases with temperature (e.g., van de Vossenberg et al., 1995). In this respect, membrane lipid composition plays a major role controlling the ion permeability of cells. At least two mechanisms have been proposed to explain the permeation of ions across lipid bilayers. The first involves a solubility-diffusion model from which permeability coefficients were calculated (Finkelstein, 1987). In many cases, however, discrepancies between predicted and measured permeability have raised questions about the validity of the solubility-diffusion concept (van de Vossenberg et al., 1995; Paula et al., 1996). An alternative mechanism has been proposed and concerns the formation of transient water wires across the membrane. These water wires can be formed spontaneously in membranes creating a pathway for proton transport by a von Grotthuß-type mechanism (Nagle and Morowitz, 1978; Deamer and Nichols, 1989; Paula et al., 1996; Haines, 2001). The extraordinary proton conductance via the von Grotthuß mechanism can result in futile ion cycling, i.e., inadvertent passage of ions across the membrane (Konings et al., 1995; van de Vossenberg et al., 1995, 1999; Valentine, 2007; Valentine and Valentine, 2009; Yoshinaga et al., 2016). Thus environmental conditions, such as temperature and pH, are expected to dictate cell membrane lipid composition (e.g., fatty acid chain length, types of lipid headgroups), which in turn controls the maintenance and dissipation of ion gradients across biological membranes (i.e., cell bioenergetics).

Several studies have shown microbial lipid adaptation to heat stress in cultured bacteria (e.g., Ray et al., 1971; Weerkamp and Heinen, 1972; Rilfors et al., 1978; Hazel and Williams, 1990) and archaea (e.g., De Rosa et al., 1980; Sprott et al., 1991; Uda et al., 2001; Matsuno et al., 2009). For instance, the important pathogen *Listeria monocytogenes* is widely known as a major foodborne disease threat. This bacterium dramatically modifies its membrane fatty acids, including longer chain fatty acids and switches from anteiso- to iso-fatty acids, when transiting from a free-living life style on refrigerated food (2–4°C) to a human pathogenic state (37°C) (Annous et al., 1997). By investigating the polar lipids of the cultured archaeon *Thermoplasma acidophilum*, Shimada et al. (2008) observed an increase in glycolipids content with increasing temperature. This result was attributed to more effective hydrogen bonds between sugar headgroups of glycolipids compared to those between glycophospho- and phospholipids (e.g., Curatolo, 1987; Baba et al., 2001).

In addition to studies using cultured microorganisms, a limited number of investigations have attempted to reconcile microbial membrane adaptations and lipid distributions in relation to elevated temperatures in natural settings. For instance, variations in the degree of cyclization of glycerol-based tetraether lipids are captured by the Ring-index, which has been applied as a proxy for archaeal membrane lipid adaptation to heat stress in terrestrial hot springs (Pearson et al., 2004, 2008; Schouten et al., 2007; Boyd et al., 2013; Paraiso et al., 2013; Wu et al., 2013; Jia et al., 2014). Supported by studies in pure cultures of thermophiles (De Rosa et al., 1980; Uda et al., 2001; Shimada et al., 2008; Boyd et al., 2011), the rationale is that by increasing the number of rings, tetraethers are packed more tightly, decreasing membrane permeability under heat stress (Gliozzi et al., 1983; Gabriel and Chong, 2000; Gliozzi et al., 2002). However, most of the studies in natural settings have demonstrated that the Ring-index may not be applicable as a universal proxy of archaeal membrane adaptation to extreme temperature ranges. Since controversial results in tetraether cyclization relative to pH were obtained in cultured thermoacidophilic archaea (Shimada et al., 2008; Boyd et al., 2011), a possible explanation for the deviation in the Ring-index and temperature can be attributed to the considerably large variation in pH and temperature at ecosystems such as hot springs (e.g., Pearson et al., 2004, 2008; Schouten et al., 2007; Jia et al., 2014).

In fact, environmental surveys attempting to probe membrane lipid adaptation or lipid/DNA biomarker distribution in relation to temperature have encountered difficulties in sampling across a thermal gradient. This is the case for sulfide deposits or black smoker chimneys in deep-sea hydrothermal vent systems. Results from lipid biomarkers and DNA evidencing a dominance of archaea vs. bacteria in the interior of chimney structures (Hedrick et al., 1992; Kormas et al., 2006) may not exclusively reflect thermal adaptation. The rationale is that the variation in temperature from 2°C (ambient seawater) to ∼350°C (chimney’s interior) may occur in less than 5 cm toward the inside of these structures (Tivey, 2007), so that interior samples are either located at inhabitable temperature zones or may record the result of seawater entrainment during sampling (Kormas et al., 2006).

**FIGURE 1:**
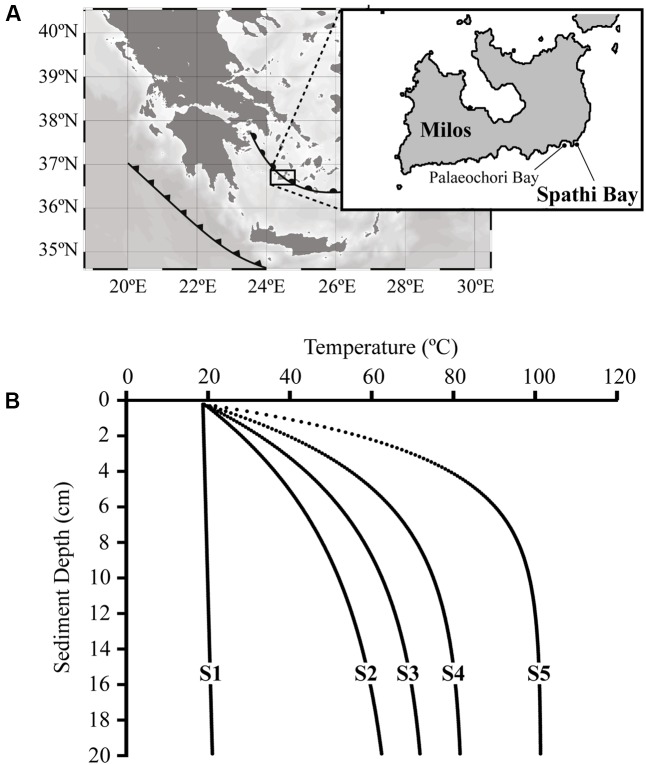
The thermal gradient studied in sediments of Spathi Bay, Milos Island, Greece. **(A)** As part of the Hellenic back-arc and fault (semi-circles and triangles, respectively), Milos Island is one of the largest hydrothermal systems of the Mediterranean Sea [modified after Giovannelli et al. (2013)]. The study site Spathi Bay is located 500 m away from Palaeochori Bay, where most geochemical and microbiological investigations from Milos Island have been performed (see Main Text). **(B)** Modeled temperature profiles in sediments of Spathi Bay calculated for every 100 μm from surface until 20 cm of sediment depth (see Materials and Methods section). Prior to sampling, stations S1 to S5 were selected based on temperatures recorded *in situ* at 5 cm sediment depth. Station S1 represents the reference sediments with temperatures below 20°C, while hydrothermally influenced stations S2 to S5 are characterized by a steep increase in temperature with depth.

In this study, we have examined a thermal gradient across a sediment field and conducted a lipidomics approach for the analysis of microbial life. For this purpose, we used a marine sediment field off the coast of Milos (Greece) featuring a point source of extreme heat generating a thermal gradient (ranging from 18 to 101°C; **Figure 1**). This thermal gradient provides ideal outdoor laboratory conditions to test how temperature drives changes in membrane lipid molecular architecture of archaea and bacteria for bioenergetic gains. That is, although considerable changes in microbial community composition are expected along this thermal gradient, we predict that any given living cell must adjust its cell membranes to *in situ* temperature conditions. We thus tested the concept that membrane lipids dictate the thermodynamic ecology of bacteria and archaea in stressful conditions (Valentine, 2007; Valentine and Valentine, 2009; Kellermann et al., 2016a). The general strategy of this paper features a detailed lipidomics approach with the focus on membrane lipid structure-function based on experimental data from the literature (e.g., pure culture experiments and/or molecular dynamics simulations).

## Materials and Methods

### Study Area

As part of the Hellenic Arc, the Milos Island is one of the largest hydrothermal systems in the Mediterranean Sea (**Figure 1A**). Extensive submarine venting is reported to occur through the sandy sediments from the intertidal zone until water depths of more than 100 m, covering an area of more than 35 km^2^ (Dando et al., 1995a, 2000). Temperatures of these sediments can be as high as 119°C (Botz et al., 1996) and the pH in pore waters slightly acidic (pH ∼5, Dando et al., 2000; Wenzhöfer et al., 2000; Price et al., 2013a). The gasses of venting fluids are characterized by high contribution of CO_2_ (55–92%), with others such as CH_4_, H_2_S, and H_2_ representing usually less than 10% each (Botz et al., 1996; Dando et al., 2000). In addition, venting fluids contain elevated concentrations of ammonium and sulfide (up to 1 mM), manganese (up to 0.4 mM) and arsenic in the micromolar range (Fitzsimons et al., 1997; Dando et al., 2000; Price et al., 2013a). Surface sediments of diffuse hydrothermal venting are covered by yellow to orange patches and white mats, which are derived from mineral deposits such as native sulfur, arsenic sulfide and/or microbial mats (Dando et al., 1998, 2000; Price et al., 2013a).

Most studies conducted in the shallow-water hydrothermal sediments off Milos Island were performed at Palaeochori Bay (e.g., Dando et al., 1995a,b, 1998; Brinkhoff et al., 1999; Sievert et al., 1999, 2000b; Giovannelli et al., 2013). Located approximately 500 m east of Palaeochori Bay (**Figure 1A**), Spathi Bay also features hydrothermal activity, including free gas emission into the water column, abundant orange to white patches in surface sediments and similar pore water geochemistry (Price et al., 2013a).

### Sampling

Sediment samples were collected by SCUBA-divers in 10 m water depth at Spathi Bay in May 2012. A permission for sample holding and processing was granted by the General Directorate of Antiquities and Cultural Heritage in Athens (permit number: Φ8/1586). The sampling site consisted of an area covered by several white patches. Before retrieving the individual sediment cores, temperature was measured *in situ* in 5 cm intervals by a handheld temperature-probe in a custom-built underwater housing (Max-Planck-Institute for Marine Microbiology in Bremen, Germany). In one of these white patches (ca. 7 m × 4 m in size), four sediment cores (up to 20 cm length), representing stations S2, S3, S4, and S5, were retrieved along a transect with increasing temperatures. An additional core was taken a few meters away from the white patches, in an area presumably free of any major vent influence, here named the reference site (S1). Sediment cores were sliced into 2 cm sections and kept at -20°C (49 samples in total for lipid analysis). For *in situ* microsensor measurements Clark-type oxygen (Revsbech and Ward, 1983), H_2_S (Jeroschewski et al., 1996) and manufactured pH microelectrodes (MI-407; Microelectrodes Inc., Bedford, NH, United States) together with an external reference (MI-401; Microelectrodes Inc., Bedford, NH, United States) were used and measurements were performed in all stations except for S2 due to weather constraints. All sensors were mounted on an autonomous profiling lander (Gundersen and Jørgensen, 1990; Wenzhöfer and Glud, 2002) deployed at the sediment-water interface. Depth profiles were recorded with a spatial resolution of 100 μm, and sensors were allowed to equilibrate at each depth for 5 s before the signal was recorded. Triplicate readings were averaged from each depth. Prior to each *in situ* measurement all microsensors were calibrated. Oxygen microsensors were calibrated by a two-point calibration, where the signal obtained in aerated seawater represented the concentration of 100% air saturation and the signal obtained in anoxic seawater (bubbled with N_2_ gas) was taken as zero oxygen. For H_2_S measurements, a 4–5 point calibration was performed in anoxic seawater of pH lower than 2. Aliquots of Na_2_S (1 M) were added stepwise into the calibration solution and the sensor signal was recorded. Subsamples, taken after each aliquot’s addition, were fixed in 2% zinc-acetate and stored at 4°C. H_2_S concentration of the subsamples was determined according to Cline (1969) by using a spectrophotometer (UV-160A Spectrophotometer, SHIMADZU GmbH, Düsseldorf, Germany). pH sensors were 2-point-calibrated using commercial buffer solutions (Mettler Toledo A.G. Analytical, Schwerzenbach, Switzerland).

### Temperature Modeling

*In situ* temperature measurements (every 5 cm) were used as input for a thermal diffusivity model run in R v. 2.9.1 (R Development Core Team, 2009)^1^ using *vegan* (Oksanen et al., 2011) and custom R scripts. For this model, we assumed a thermal diffusivity of 3 × 10^-7^ m^2^ s^-1^ (for sandy sediments) and steady flow velocity of 0.021 m h^-1^. Temperature values for sediment sections of 100 μm were determined. Temperature values for S2 were predicted based on bottom water temperature and a single *in situ* value (∼40°C) obtained at 5 cm sediment depth. Fluid flow velocity and diffusivity were modeled for S2 based on the values obtained for the hydrothermal influenced sediments at stations S3 to S5 (**Figure 1B**).

### Lipid Extraction

Lipids were extracted from 30 to 60 g of freeze-dried sediments using a modified Bligh and Dyer method as in Sturt et al. (2004). In brief, an internal standard 1,2-dihenarachidoyl-sn-glycero-3-phosphocholine (*C*_21:0_/*C*_21:0_–PC, Avanti Lipids) and a mixture of dichloromethane/methanol/buffer (DCM/MeOH/buffer, 1:2:0.8; v/v/v) was added to the sediment and ultrasonicated for 10 min in four steps. For the first two extraction steps a phosphate buffer was used (pH 7.4), and, for the last two steps, a trichloroacetic acid buffer (50 g/L, pH 2.0). After each ultrasonication, samples were centrifuged and the supernatant collected in a separatory funnel. For phase separation equal amounts of DCM and deionized Milli-Q water were added to a final volume of 1:1:0.8 (MeOH/DCM/buffer, v/v/v). The organic phase was separated and the remaining aqueous phase washed three times with DCM. Subsequently the DCM phase was washed three times with deionized Milli-Q water, evaporated close to dryness under a stream of nitrogen at 37°C and stored at -20°C as total lipid extract (TLE) until further analysis.

### Analyses and Quantification of Polar Lipids

An aliquot of each TLE was analyzed for polar lipid quantities on a Dionex Ultimate 3000 high performance liquid chromatography (HPLC) system connected to a Bruker maXis Ultra-High Resolution quadrupole time-of-flight tandem mass spectrometer (qTOF-MS) equipped with an ESI ion source (Bruker Daltonik, Bremen, Germany). Polar lipid analyses by HPLC-MS were performed using three different methods: normal and reverse phase following the protocol of Wörmer et al. (2013) to quantify non-archaeal and archaeal polar lipids, respectively; and reverse phase according to Zhu et al. (2013) for ring, unsaturation, methylation indices for both core and polar archaeal tetraethers. Detection of lipids was performed in positive and/or negative ionization mode while scanning a mass-to-charge (m/z) range from 150 to 2,000. MS^2^ scans were obtained in data-dependent mode, for each MS full scan up to three MS^2^ experiments were performed, targeting the most abundant ions. Active exclusion limits the times a given ion is selected for fragmentation (three times every 0.5 min) and thus allowed to also obtain MS^2^ data of less abundant ions. Lipid identification was achieved by monitoring exact masses of possible parent ions (present as either H^+^, NH_4_^+^, or Na^+^ adducts) in combination with characteristic fragmentation patterns as outlined by Sturt et al. (2004) and Yoshinaga et al. (2011). Polar lipid quantification was performed by comparison of parent ion responses relative to known amounts of an internal standard (i.e., *C*_21:0_/*C*_21:0_–PC) and normalized to gram of dry sediment weight. Reported concentrations were corrected for response factors using commercially available or purified standards (see Supplementary Table S1). Additional analyses of archaeal core tetraethers, which lack a polar headgroup, were performed according to Becker et al. (2013).

### Correlation Analysis

Given that the data points were not normally distributed, we conducted a two-sided Spearman’s rank correlation test for several archaeal and non-archaeal lipid parameters against temperature. Spearman’s rank correlations were calculated using the software of Wessa (2017). Parameters included the total number of individual archaeal and non-archaeal polar lipids from each sample, which was defined as lipid diversity. For bacterial polar lipids, we calculated the averaged number of unsaturation(s) and chain length of lipids from specific compound classes (e.g., SQ, CL, and PC). That is, the number of double bonds and chain length of each individual lipid were multiplied by the relative abundance within a compound class and summed up. For details in core and polar archaeal tetraether indices calculation (number of cyclopentane rings, degree of unsaturation and additional methyl groups) please see the Supporting Information.

## Results

### The Thermal Gradient in Sediments from Spathi Bay

**Figure 1B** displays the modeled temperature profiles from surface sediments until 20 cm sediment depth of stations S1–S5. Temperature profiles from diffuse hydrothermal venting stations showed a uniform trend of continuous and steep increasing values within the first centimeters and a flattened slope at greater depths. For instance, station S5 displayed the strongest temperature gradient (ΔT > 80°C from top to bottom) and the highest temperature recorded in Spathi sediments (101°C at 20 cm sediment depth). The temperature of the reference site S1 was relatively constant throughout the core, increasing only slightly from 18°C at surface to 20°C at 20 cm sediment depth.

This thermal gradient in Spathi sediments is generated by the venting fluids as evidenced in the *in situ* profiles of pH and H_2_S recorded by microsensors (Supplementary Figure S1). As a remarkable characteristic of the influence of venting fluids in sediments with temperatures >20°C, we observed a downcore decrease in pH (from seawater pH of ∼8.4 to ∼5.5) and an increase in H_2_S concentrations (up to ca. 2 mM; Supplementary Figure S1). The investigated sediments displayed an abrupt decrease in oxygen concentration to anoxic conditions within the first millimeters (Supplementary Figure S1). Oxygen depletion within the first cm of sediments was observed in all stations, including S1, indicating that sediments analyzed in this study were mostly associated with anoxic conditions.

### Polar Lipid Distribution along a Thermal Gradient in Marine Sediments

**FIGURE 2:**
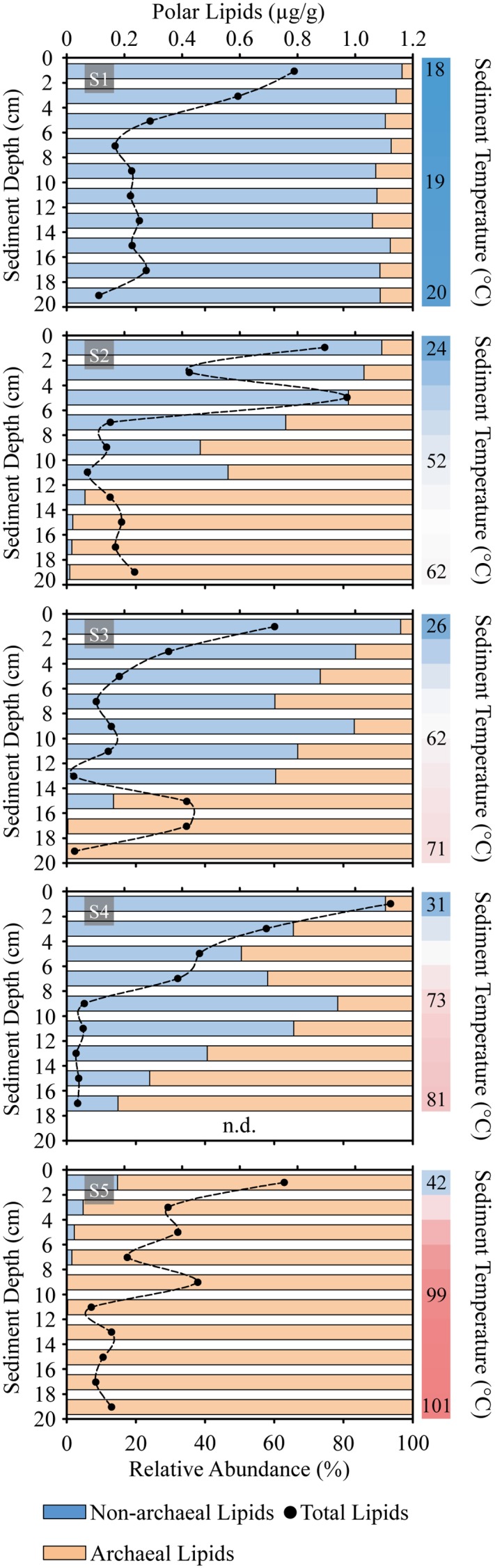
Sediment profiles of concentration of total microbial polar lipids (dots) and distribution of archaeal vs. non-archaeal lipids (horizontal bars) in Spathi Bay. Samples were analyzed in 2 cm horizons from surface until 20 cm of sediment depth. Adjacent vertical bars indicate the thermal gradient of each station with upper, mid and bottom sediments temperatures in °C. n.d., no data.

With some exceptions, total polar lipid content in the sediments decreased downcore (**Figure 2**). Averaged concentrations of polar lipids were highest at S2, S4, and S1 (>0.31 μg g^-1^), followed by S5 and S3. The highest total polar lipid concentration of >1.1 μg g^-1^ sediment was recorded at the surface layer of S4, whereas the lowest concentration was detected at S3 (12–14 cm) with 0.025 μg g^-1^. While the relative abundance of non-archaeal lipids was highest at the reference site (S1) and in surface layers from hydrothermally influenced sediments (in general <12 cm), the contribution of archaeal lipids increased with temperature of the sediments (i.e., downcore and toward S5).

### Archaeal Polar Lipid Distribution

**FIGURE 3:**
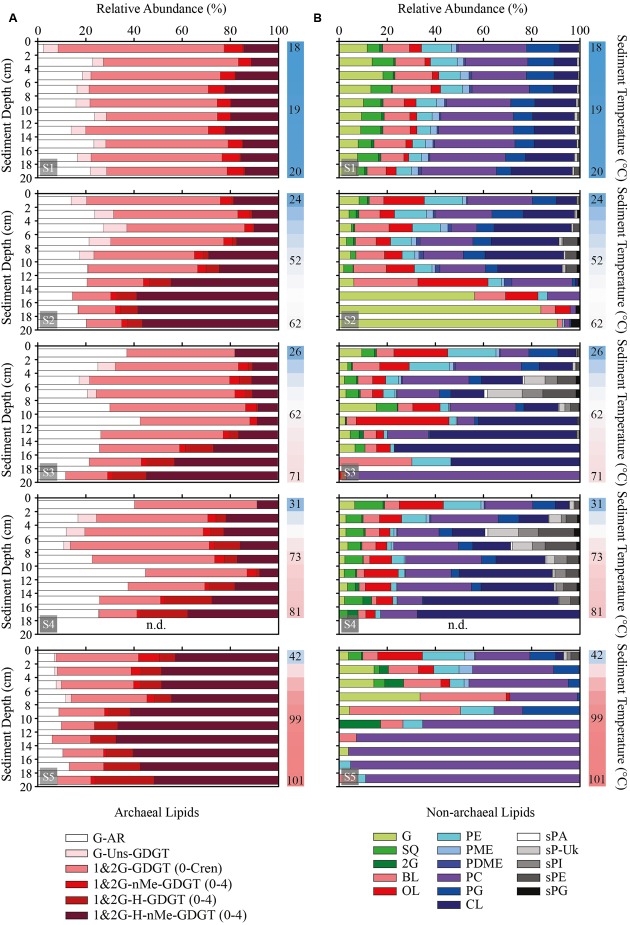
Sediment profiles displaying the abundance of polar lipid classes relative to total archaeal **(A)** and total non-archaeal **(B)** lipids. Note that this figure should be interpreted together with the relative abundance of archaeal and bacterial polar lipids shown in **Figure 2**. Samples were analyzed in 2 cm horizons from surface until 20 cm of sediment depth. Adjacent vertical bars indicate the thermal gradient of each station with upper, mid and bottom sediments temperatures in °C. n.d., no data. Abbreviations: monoglycosidic archaeol (AR); monoglycosidic unsaturated (Uns) acyclic glycerol-dibiphytanyl-glycerol-tetraethers (GDGT); mono- and diglycosidic GDGT with 0 to 5 rings (0-Cren); mono- and di-glycosidic GDGT with up to 4 rings divided into mono- and di-methylated (nMe), H-shaped (H) and H-nMe-GDGT with up to 4 methyl groups. Cren, crenarchaeol. For abbreviations of non-archaeal lipid classes please see Main Text. sP-Uk, unknown glycosylated phosphatidyl sphingolipids.

Archaeal polar lipids were identified and quantified by high performance liquid chromatography coupled with mass spectrometry (HPLC-MS). Archaeal polar lipids consisted exclusively of glycosidic head groups and were categorized in six classes according to their isoprenoidal side chains characteristics (see Supplementary Figure S2). Polar lipids included a monoglycosyl archaeol (G-AR) as the only archaeal diether and a variety of different glycerol-dibiphytanyl-glycerol-tetraethers (GDGT) with monoglycosyl (G) and minor contributions of diglycosyl (2G) headgroups. Notably, archaeal polar lipids were dominated by tetraethers over diethers (**Figure 3A**). Archaeal tetraethers included acyclic to pentacyclic GDGT (GDGT-0 to -Cren, or caldarchaeol to crenarchaeol), unsaturated tetraethers (Uns-GDGT, with up to four double bonds), GDGT with a covalent bond linking the two biphytanyl chains into an H-shaped structure (H-GDGT), and presence of one to four additional methyl groups (nMe) in the isoprenoidal side chains (both nMe-GDGT and H-nMe-GDGT). H-GDGT and H-nMe-GDGT consisted of acyclic to tetracyclic compounds.

**FIGURE 4:**
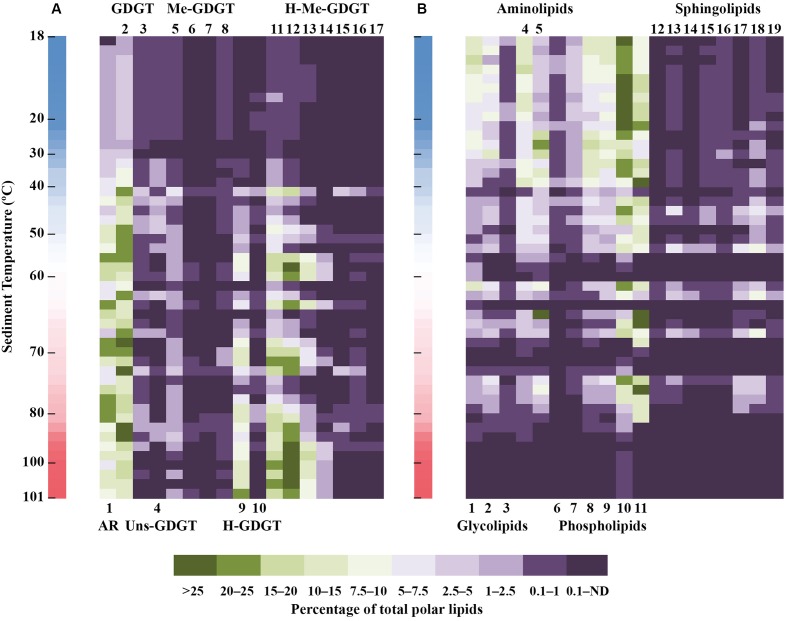
Heat map distribution of archaeal **(A)** and non-archaeal **(B)** polar lipid classes in sediments of Spathi Bay. Green to dark purple scale (in %) refers to the contribution of lipid classes relative to the total polar lipid concentration. ND, not detectable. Adjacent vertical bars indicate the thermal gradient in sediments ranging from 18 to 101°C. List of compounds in A (archaeal lipids): 1 (G-AR), 2 (G-GDGT), 3 (2G-GDGT), 4 (G-Uns-GDGT), 5 (G-Me-GDGT), 6 (G-2Me-GDGT), 7 (2G-Me-GDGT), 8 (2G-2Me-GDGT), 9 (G-H-GDGT), 10 (2G-H-GDGT), 11 (G-H-1Me-GDGT), 12 (G-H-2Me-GDGT), 13 (G-H-3Me-GDGT), 14 (G-H-4Me-GDGT), 15 (2G-H-1Me-GDGT), 16 (2G-H-2Me-GDGT), 17 (2G-H-3Me-GDGT). List of compounds in B (non-archaeal lipids): 1 (G), 2 (SQ), 3 (2G), 4 (BL), 5 (OL), 6 (PDME), 7 (PME), 8 (PE), 9 (PG), 10 (PC), 11 (CL), 12 (sPA), 13 (sP-Uk1), 14 (sP-Uk2), 15 (sP-Uk3), 16 (sP-Uk4), 17 (sPI), 18 (sPE), 19 (sPG). sP-Uk1–4: unknown glycosylated phosphatidyl sphingolipids (see Supplementary Figure S3 for structure identification).

Monoglycosidic AR and GDGT were the most widely distributed archaeal polar lipids with highest contribution between 40 and ∼90°C (**Figure 4A**). With highest relative abundance associated with temperatures above 50°C, the monoglycosidic H-GDGT, H-1Me- and H-2Me-GDGT were also abundant archaeal lipids in the sediments of Spathi Bay. Particularly interesting are the high contributions of monoglycosidic H-2Me-GDGT in sediments above 80°C. In sediments with temperatures below 40°C, other abundant archaeal lipids included monoglycosidic Me-GDGTs and diglycosidic forms of H-GDGT and H-nMe-GDGTs. In contrast with these lipids, the distribution of unsaturated tetraethers was mostly associated with sediments displaying temperatures between 30 and 50°C.

### Non-archaeal Polar Lipid Distribution

Non-archaeal polar lipids can be divided into four major groups: glycolipids, phospholipids, aminolipids, and sphingolipids (Supplementary Figure S2). Glycolipids and phospholipids were exclusively identified as diacylglycerol lipids (DAG), i.e., the side chains were represented by two fatty acids. A variety of phospholipids were detected in the sediments, including phosphatidyl-ethanolamine, -*N*-methylethanolamine, -*N*-dimethylethanolamine, -glycerol and -choline (PE, PME, PDME, PG, and PC, respectively) and cardiolipin (CL). The glycolipids were composed of sulfoquinovosyl (SQ), mono and diglycosyl (G and 2G) headgroups, and aminolipids consisted of ornithine and betaine lipids (OL and BL). The sphingolipids were composed exclusively of phosphatidyl-based headgroups such as ethanolamine (sPE), glycerol (sPG), phosphatidic acid (sPA), and inositol (sPI), with the latter two headgroups not observed among the DAG phospholipids (**Figures 3B**, **4B**). Based on HPLC-MS experiments in comparison with sphingomyelin, we tentatively identified the side chains of these sphingolipids with a terminal methyl group (Supplementary Figure S3) as reported for Bacteroidetes and *Sphingobacterium* (Olsen and Jantzen, 2001; Naka et al., 2003; An et al., 2011; Wieland Brown et al., 2013).

A general trend of decreasing concentrations with increasing temperature was observed for several DAG lipids including glycolipids, BL, PDME, PME, PE, and PG (**Figure 4B**). In contrast to this distribution, the sphingolipids were mostly associated with sediment temperatures ranging from 30 to 80°C. Among the aminolipids, the highest contribution of OL occurred between 25 and 50°C with peaks in concentration associated with sediments as hot as 80°C. Although displaying highest contribution in sediments below 30°C, PC together with CL were the major DAG lipids in sediments with temperatures between 60 and 85°C. In these relatively “hot” sediments, CL and PC displayed contributions with percentages as high as the most abundant archaeal lipids (**Figure 4**).

### Molecular Species of Polar Lipids Revealed Trends in Relation to Sediment Temperature

**FIGURE 5:**
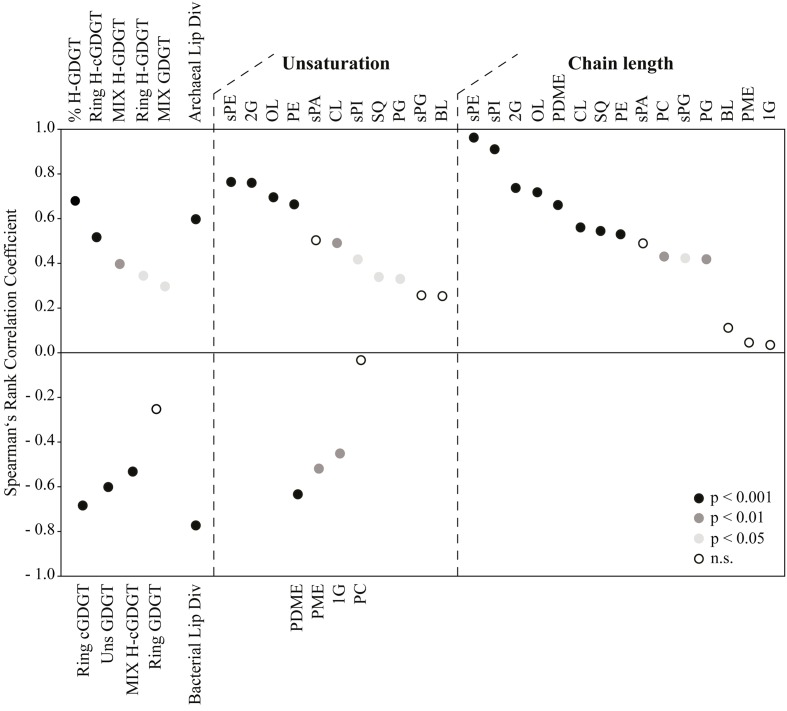
Analysis of molecular species of archaeal and non-archaeal lipids as a function of temperature. Spearman’s rank correlation coefficient values corresponding to positive or negative correlation (see Materials and Methods and Supplementary Table S2) between lipid parameters and temperature are displayed as circles, with colors indicating the level of significance (*p* < 0.001, *p* < 0.05, *p* < 0.01 or not significant, n.s.). Archaeal/Bacterial Lip Div, archaeal/bacterial lipid diversity. Please see Supporting Information for details about lipid parameters used as input to the correlation analysis.

In order to access the information encoded in the assemblage of polar lipids, we examined a Spearman’s rank correlation between several lipid indices and temperature (**Figure 5**, see Supporting Information for detailed calculations of indices). As already evidenced in **Figure 4A**, the percentage of H-GDGT (including the H-nMe-GDGT) was positively correlated with temperature (*p*-value: 2.37e-07). We also observed a positive correlation between temperature and number of additional methyl groups in tetraethers (MIX-index for H-GDGT/GDGT) and number of cyclopentane rings in H-GDGT (Ring-index for both polar and core lipids). Other parameters such as the Ring and Unsaturation (Uns) indices for core and polar GDGT as well as MIX for core H-GDGT were negatively correlated with temperature while the weak negative correlation of the Ring index for polar GDGT was not significant (*p*-value: 8.39e-02). Furthermore, our data revealed that the diversity of archaeal lipids (estimated as the number of individual lipids in a given sample) increased with temperature whereas non-archaeal lipids displayed an opposing trend (**Figure 5**). Interestingly, for most non-archaeal lipids we observed a significant positive correlation between temperatures and degrees of unsaturation and chain length. Only a few DAG lipid classes such as G, PME, and PDME displayed a significant negative correlation between the Uns-index and temperature. The exact calculated two-sided *p*-values and the individual values of the Spearman’s rank correlation coefficient can be found in Supplementary Table S2.

## Discussion

### Environmental Setting and Potential Sources of Polar Lipids in Sediments of Spathi Bay

The studied sediments of Spathi Bay covered a broad temperature range of 18–101°C, at the high end approaching the currently determined limit of life (∼120°C; Kashefi and Lovley, 2003; Takai et al., 2008). Temperature gradients were observed vertically in downcore profiles as well as horizontally on the transect from S1 to S5 (**Figure 1**). These conditions are also observed at other sites of Milos Island, such as at Milos, Boudia, and Palaeochori bays (Dando et al., 1995a; Fitzsimons et al., 1997; Wenzhöfer et al., 2000; Aliani et al., 2004; Price et al., 2013a; Yücel et al., 2013). Hydrothermally affected sediments off Milos Island are characterized by steep geochemical gradients, and might undergo daily fluctuations by waves, tides, and seismic events that influence the intensity of discharged fluids (Aliani et al., 2004; Yücel et al., 2013). Steep geochemical gradients included not only suboxic to anoxic transition within the first millimeters, but also lower pH and higher H_2_S concentrations toward the source fluid, i.e., downcore (Supplementary Figure S1). In addition to reporting the influence of source fluids, we suggest that temperature is one of the key factors controlling microbial populations in sediments of Spathi Bay, as it has been reported for other hydrothermal environments (Schrenk et al., 2008; Boyd et al., 2013; Cole et al., 2013; Sharp et al., 2014). Furthermore, the highest concentrations of arsenic in the marine environment have been reported for sediments off Milos (Price et al., 2013a), indicating that source fluids are not only “hot,” but also toxic. In order to cope with frequent changes in temperature, and consequently in toxic conditions, bacteria and archaea inhabiting these sediments must continuously adapt their cell membrane lipid composition.

The identified glycosidic diethers and tetraethers are widespread among archaea (e.g., Kates, 1993; Koga et al., 1993). However, several of these lipid features are characteristic of thermo- and/or hyperthermophilic archaea, which are known to inhabit the sediments of Milos (e.g., Thermococcales, Archaeoglobales, and *Thermoprotei*; Dando et al., 1998; Price et al., 2013b). The “thermophilic” lipid features appeared exclusively in tetraethers (see Supplementary Figure S2) and included additional methylation(s) in isoprenoidal chains as found in *Methanothermobacter thermautotrophicus* (Knappy et al., 2009; Yoshinaga et al., 2015a) and *Thermococcus kodakarensis* (Meador et al., 2014), H-shaped configuration as in *Methanothermus fervidus* (Morii et al., 1998), *Aciduliprofundum boonei* (Schouten et al., 2008), and *Ignisphaera aggregans* (Knappy et al., 2011), or a combination of both as in *Methanopyrus kandleri* (Liu et al., 2012). Thus far, these modified tetraethers have only been detected as major archaeal lipids at hydrothermally influenced systems such as deep-sea vents and hot springs (e.g., Jaeschke et al., 2012; Lincoln et al., 2013; Gibson et al., 2013; Schubotz et al., 2013; Reeves et al., 2014b; Jia et al., 2014).

Archaeal polar lipids possess high preservation potential in marine sediments (Schouten et al., 2010; Logemann et al., 2011; Xie et al., 2013). In fact, several archaeal lipids that we attribute to thermophilic and/or hyperthermophilic archaea (e.g., H-GDGT, H-nMe-GDGT) were also found in the reference core S1 with temperatures below 20°C (**Figures 3A**, **4A**). Moreover, regular GDGTs (i.e., 1&2G GDGT 0-Cren) that are dominant archaeal polar lipids in non-hydrothermal marine sediments (Lipp and Hinrichs, 2009; Schouten et al., 2013) were found throughout the transect and at all depths. Although thermophilic Thaumarchaeota may represent potential sources of GDGT-Cren, these archaea have not been detected in 16S rRNA gene libraries from Milos sediments (Dando et al., 1998; Price et al., 2013b) and are not considered major players in marine hydrothermal settings (de la Torre et al., 2008; Pitcher et al., 2010; Sinninghe Damsté et al., 2012). We thus conclude that archaeal polar lipids in the hydrothermally heated sediments of Spathi Bay may partially reflect records of past communities – both from *in situ* production (in the case of H-GDGT or H-nMe-GDGT) and the water column (particularly the GDGT-Cren). Such a preservation scenario for archaeal lipids has been also reported for an inactive sulfide deposit in Manus Basin deep-sea hydrothermal system (Reeves et al., 2014b).

There are at least two fundamental aspects that limit eukaryotic life in the thermophilic sediments of Milos: temperatures higher than 60°C (e.g., Brock, 1967; Tansey and Brock, 1972; Rothschild and Mancinelli, 2001) and predominance of anoxic conditions after the first few millimeters in the sediments (Supplementary Figure S1). Although abundantly distributed in surface sediments of Milos, photosynthetic algae and cyanobacteria are only expected at the sediment-water interface and were reported from several studies as brownish or green tinge on top of the sediments or on top of white mats, which cover hydrothermally active sediments (Dando et al., 1995b, 2000; Thiermann et al., 1997; Sartoni and De Biasi, 1999; Sievert et al., 2000a). Photosynthetic membranes of algae and cyanobacteria are enriched in glycolipids such as G-, 2G-, and SQ-DAG (e.g., Guschina and Harwood, 2006; Wada and Murata, 2009). If photosynthetic organisms were major sources of polar lipids in surface sediments one could expect drastic changes in lipid composition from surface sediments to anoxic sediments at the reference site S1. Nevertheless, the polar lipids distribution at S1 was largely uniform throughout the sediment core (**Figure 3B**). Although there exists the possibility that these glycolipids represent fossil material, analysis of acyl chains of BL, another well-known component of photosynthetic membranes (Dembitsky, 1996; Kato et al., 1996), revealed the presence of odd chain fatty acids (e.g., *C*_15:0_ and *C*_17:0_) typical of bacteria (Schubotz et al., 2009).

Among eukaryotes, nematodes and fungi are also potential sources of phospholipids and sphingolipids in the anoxic sediments of Milos, as they have been commonly found associated with hydrothermal ecosystems (e.g., Dando et al., 1995b; Thiermann et al., 1997; Le Calvez et al., 2009; Burgaud et al., 2014; Portnova et al., 2014). Whereas phospholipids such as PC and CL (**Figure 3**) are characteristic of mitochondria (Horvath and Daum, 2013), eukaryotic membranes are enriched in sugar-based ceramides and sphingomyelin (Hannun and Obeid, 2008; Merrill, 2011). The latter lipids were not apparent in our samples, instead sphingolipids in Milos sediments were dominated by uncommon phosphate-based headgroups such as PE, PG, and PI (**Figure 3** and Supplementary Figure S3). Our results thus suggest that bacteria rather than eukaryotes are likely the major sources of non-archaeal polar lipids throughout the sediments of Spathi Bay. Therefore the non-archaeal lipids are referred as mostly bacterial in origin throughout the following discussion.

The bacterial polar lipid distribution according to head groups in surface sediments at S2 to S5 was generally comparable to those at S1 (**Figure 3B**), indicating mesophilic bacteria as likely sources of lipids. In contrast to S1, the hydrothermally influenced sediments at S2 to S5 revealed significant downcore shifts in polar lipid distribution, which we attribute to the presence of thermophilic bacteria. Data on 16S rRNA gene libraries revealed dominance of mesophilic *Epsilonproteobacteria* in surface sediments of Palaeochori Bay, with other minor groups composed of Cytophaga-Flavobacteria-Bacteroidetes, *Gammaproteobacteria* and *Deltaproteobacteria* (Sievert et al., 2000a; Giovannelli et al., 2013; Price et al., 2013b). The few 16S rRNA-gene based phylogenetic studies that investigated sediments deeper than 2 cm reported considerable shifts in bacterial community composition relative to the surface sediments, notably the presence of Bacilli, Planctomycetes and thermophilic bacteria such as *Thermodesulfobacteria*, *Thermomicrobia* and *Thermotogae* (Sievert et al., 2000a; Price et al., 2013b).

Lipid biomarkers have proven to be important tools for monitoring microbial ecology of marine environments, where most species of archaea and bacteria are uncultured (e.g., Hedrick et al., 1992; Hinrichs et al., 1999; Schubotz et al., 2009; Kellermann et al., 2012; Gibson et al., 2013; Lincoln et al., 2013; Reeves et al., 2014b). In some cases, however, polar lipids lack the taxonomic specificity of DNA-based techniques. For instance, major DAG phospholipid classes identified in this study such as CL, PE, and PG are common among cultured bacteria (e.g., Goldfine, 1984; Dowhan, 1997; Sohlenkamp et al., 2003), including *Epsilonproteobacteria* that are widespread in surface sediments of Milos. Others, more specific polar lipids such as sphingolipids, may be assigned to Sphingobacteria or Bacteroidetes (e.g., Kato et al., 1995; Olsen and Jantzen, 2001), which are detected by 16S rRNA-gene sequencing in Milos sediments (Price et al., 2013b). While abundant in sediments, archaeal H-nMe-GDGT have been exclusively described for the hyperthermophilic *M. kandleri* (Liu et al., 2012), which is not apparent in archaeal 16S rRNA-gene surveys of Milos (Dando et al., 1998; Price et al., 2013b). Rather than applying lipids as chemotaxonomic markers, we attempted to reconcile microbial membrane adaptations based on polar lipid distribution along a thermal gradient in sediments of Spathi Bay.

### Archaeal Polar Lipid Quandary: Does the Dominance of Archaeal Lipids in Sediments with Elevated Temperatures Reflect the Extremely Low Permeability of Their Membranes?

The thermal gradient sampled in sediments of Spathi Bay (**Figure 1**) allows inferring membrane lipid adaptation to temperature. Our results evidenced a higher contribution of archaeal lipids compared to bacterial lipids in deep layers of the sediments influenced by hydrothermal fluids (**Figures 2**, **4**). As discussed earlier, a preservation scenario could explain the dominance of archaeal vs. bacterial lipids at elevated temperatures. However, the significant correlation of “thermophilic” H-GDGT and H-nMe-GDGT as polar lipids with increasing temperatures (% H-GDGT in **Figure 5**) suggests active communities as the most important source of lipids in high temperature sediments. Furthermore, addition of cyclopentane rings and/or methyl groups to these archaeal H-shaped tetraethers, which are proposed to decrease membrane permeability (see Discussion below), appeared to be significantly correlated with temperature. Thus, in agreement with Valentine (2007), we propose that the dominance of archaeal polar lipids in sediments with elevated temperatures reflects the generally lower ion permeability relative to the bacterial analogs. It is important to note that in Milos sediments, hydrothermal fluid advection is associated with high temperatures and accompanying toxic compounds such as arsenic (Price et al., 2013a). Thus membrane lipid adaptations may likely reflect the extent of these conditions.

Archaeal polar lipids detected in sediments of Spathi Bay were exclusively glycolipids. Glycolipid-rich membranes are proposed to be more resistant to temperature and low pH than phospholipids given the tight hydrogen bonding between sugar headgroups (Curatolo, 1987; Baba et al., 2001; Shimada et al., 2008; Wang et al., 2012). Another interesting feature of archaeal lipids observed in sediments of Spathi Bay was the dominance of tetraethers over diethers (**Figure 3A**). Controlled experiments using cultured thermophilic archaea such as methanogens (Sprott et al., 1991), *Thermococcus* (Matsuno et al., 2009) and *Archaeoglobus fulgidus* (Lai et al., 2008) have shown that the di- to tetraether ratio tends to decrease with increasing temperature. According to these data, a higher proportion of tetraether lipids over diether lipids in archaeal membranes represents a clear lipid adaptation to elevated temperatures. Although a tetraether structure itself confers low ion permeability to the membrane (Koyanagi et al., 2016), the modifications of archaeal tetraether structures revealed by our study (e.g., H-shape, additional methylation or cyclopentane rings) suggest further requirements for avoiding futile ion cycling or ion leakage in high-temperature environments.

Increasing sediment temperatures correlated with increased proportions of H-shaped GDGT and tetraethers with additional methyl groups in biphytane chains, i.e., nMe-GDGT and H-nMe-GDGT (**Figures 4**, **5**). Although these features of archaeal tetraethers have been exclusively found in cultured thermophilic archaea (Morii et al., 1998; Sugai et al., 2004; Schouten et al., 2008; Knappy et al., 2009, 2011; Liu et al., 2012; Yoshinaga et al., 2015a), little is known about their functions in the membranes. For instance, it is generally accepted that H-shaped GDGT represent an archaeal membrane adaptation to heat stress (e.g., Morii et al., 1998). We borrow from Thomas Haines (Haines, 2001) the concept of membrane bulking, which predicts that lipid structures such as fatty acids with additional methyl groups, sterols or squalane can crowd the hydrophobic region of bilayers making them bulkier. The net effect is that these lipid structures may effectively prevent formation of water clusters in the lipid bilayer (see Haines, 2001), thus avoiding ion leakage across the membrane. In this sense, H-shaped GDGT may reduce the fluidity of biphytanyl chains, thereby stabilizing van der Waals forces among isoprenoidal chains from adjacent lipids. In analogy to the functions of methyl-branched fatty acids in bacteria (e.g., Suutari and Laakso, 1992; Haines, 2001), additional methyl groups in biphytane(s) of tetraethers may provide extra bulking between neighbor membrane lipids, thus reducing ion permeability of archaeal membranes under heat stress. In addition to membrane bulking, recent experiments with molecular dynamics simulations revealed that methyl branching enhances membrane fluidity of membranes composed of fatty acids (Poger et al., 2014). This effect may thus also hold true for methylated tetraethers of archaea in Spathi sediments.

The presence of cyclic biphytanes is proposed to reduce membrane thickness while leading to stronger interaction between neighbor isoprenoidal chains (Gabriel and Chong, 2000; Gliozzi et al., 2002). The absence of significant correlation between increasing number of rings in regular GDGT (0-Cren) with temperature (**Figure 5**) contrasts with laboratory experiments using cultured thermoacidophilic archaea (Shimada et al., 2008; Boyd et al., 2011; Jensen et al., 2015). Interestingly, the later studies reported diverging responses of tetraether cyclization relative to pH conditions. This observation in combination with our results suggest that cyclization of archaeal tetraether might reflect a net effect of several parameters important for bioenergetics rather than only membrane bulking for thermal adaptation. We consider three possibilities to explain our findings. First, that archaea producing regular GDGT (0-Cren) do not respond to increasing temperature by increasing the number of rings in their biphytanyl chains. Second, that active thermophilic archaea producing H-GDGT and H-nMe-GDGT increase the number of rings with temperature (**Figure 5**), whereas regular GDGT are mainly sourced by less active or dead archaea (see preservation scenario above). Third and the one that we favor: cyclopentane rings may not be an exclusive membrane permeability response to temperature.

**FIGURE 6:**
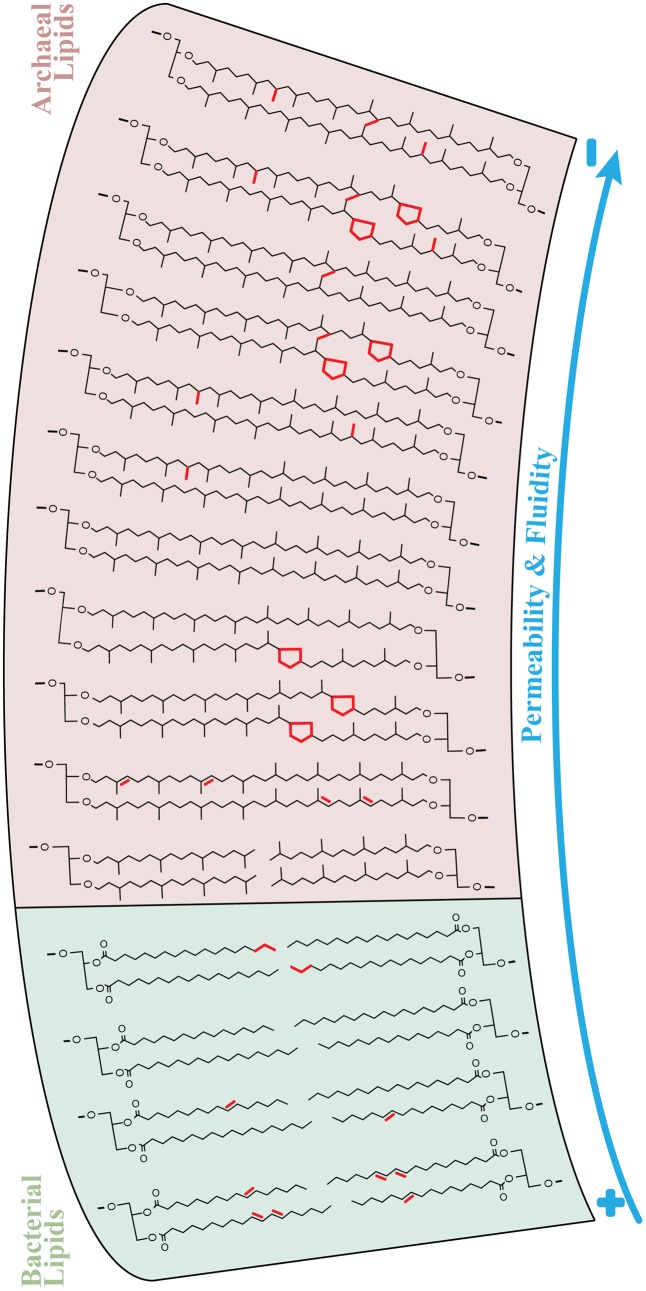
Lipid chemical structures determining ion permeability (e.g., proton and sodium) and fluidity of archaeal and bacterial membranes in sediments of Milos. The arrows indicate a continuum trend from high (+) to low (–) membrane permeability and fluidity. The essential structural distinction between archaeal and bacterial lipid membranes are the ether-linked isoprenoids in the former and typical ester-linked fatty acids in the latter. The permeability and fluidity properties of bacterial membranes were mainly regulated by the degree of unsaturation (i.e., number of double bonds) and chain length of fatty acids (highlighted in red), as shown in **Figure 5**. Apart from the transition diether to tetraether, i.e., bilayer to monolayer membranes, archaea featured several modifications in tetraethers that are suggested to influence the permeability and fluidity properties of their membranes (**Figure 5**). These features (in red) include unsaturation, cyclization and methylation of biphytane(s) and covalent bonds between isoprenoidal chains (H-shaped).

One fundamental aspect to consider is that the presence of methyl groups protruding from isoprenoidal side chains is the main reason explaining the higher stability of archaeal compared to bacterial lipids (e.g., Degani et al., 1980; Yamauchi and Kinoshita, 1993; Yamauchi et al., 1993; Elferink et al., 1994; Baba et al., 2001; Mathai et al., 2001). Although the addition of cyclopentane rings has been proposed to decrease permeability in molecular dynamics simulations (MDS; Gabriel and Chong, 2000; Gliozzi et al., 2002), the presence of rings corresponds to a one-to-one loss of methyl groups in isoprenoidal tetraethers. Accordingly, Sinninghe Damsté et al. (2002) comparing GDGT-4 and -Cren in MDS, concluded that the latter provided less dense packing of biphytanyl chains likely resulting in a lower thermal transition point, i.e., increased fluidity. Therefore, we alternatively suggest that the presence of cyclopentane rings in archaeal tetraethers could dramatically increase membrane fluidity/motion while keeping isoprenoidal chains neighbors compacted in the hydrophobic environment. In support of our argumentation, recent MDS experiments have evidenced a dual role of cyclopropane fatty acids in stabilizing membranes and promoting their fluidity, which in general terms is distinct from the analogous unsaturated chains (Poger and Mark, 2015). Note that this “double-function” of cyclopentane rings in tetraethers is slightly different than that of mobile, but less compacted unsaturated biphytanes (**Figure 6**).

In summary, the dominance of archaeal over bacterial polar lipids in sediments with high temperatures indicates that membrane lipids may be directly linked to the higher potential for energy conservation of archaea compared to bacteria under stress conditions (Valentine, 2007; Valentine and Valentine, 2009). Alternatively, this finding may reflect the high preservation potential of archaeal lipids in marine sediments (Schouten et al., 2010; Xie et al., 2013), i.e., lower degradation rates of lipids derived from archaea compared to bacteria (Logemann et al., 2011). Our data, however, support the idea that the high abundance of H-GDGT and H-nMe-GDGT in sediments with elevated temperatures is related to the low permeability properties induced by H-shape and additional methyl groups of archaeal tetraethers. Moreover, cyclic tetraethers and/or extra methyl group(s) may provide both increased fluidity/motion and reduced permeability for archaeal membranes. Finally, we cannot exclude that these adaptations at the archaeal cell membrane level may also be used as mechanisms to cope with other stresses, such as low pH, high sulfide and arsenic concentrations that are commonly encountered in the hydrothermally heated sediments of Spathi Bay (Supplementary Figure S1; Price et al., 2013a; Gomez-Saez et al., 2016).

### Bacterial Lipids Quandary: Benefit and Risk of Membrane Fluidity and Futile Ion Cycling

Bacterial polar lipids concentrations decreased abruptly with increasing temperature (**Figures 2**, **4B**). A dominance of bacterial phospholipids even under the highest measured temperatures introduces a controversial picture to the energy conservation properties of archaeal glycolipids (see above). We suggest two explanations that may account for the dominance of phospholipids among bacteria. The first involves energy invested in the synthesis of complex envelope structures (e.g., outer membrane, peptidoglycan layers) that would provide a robust permeability barrier at high temperatures. The rationale is that H-bonded lipopolysaccharides of Gram-negative bacteria, e.g., lipid A (Nikaido, 2003), or several wraps of peptidoglycan layers and associated structures in cyanobacteria or Gram-positive bacteria (Ward et al., 1998; Bansal-Mutalik and Nikaido, 2014), would provide an effective protection for cells under heat stress. Recall that archaeal cell envelopes are less sophisticated than bacterial cell envelopes (Albers and Meyer, 2011), and likely explain the dominance of sugar headgroups as a hallmark of archaeal lipids in hydrothermally heated sediments of Spathi Bay. The second explanation concerns lipid adaptations at the side chain level and/or by increasing amounts of membrane-stabilizing lipids such as sphingolipids.

For most of the polar bacterial DAG lipids, we observed a trend of increased average chain length with increasing temperature (**Figure 5**). Data on lipid vesicles have shown that polar lipids composed of longer fatty acids in their side chains are less permeable to ions, including H^+^, K^+^, and Cl^-^, than the ones linked to shorter fatty acids (Paula et al., 1996). In addition, there is robust experimental evidence suggesting that polar lipid bilayers become thinner with increasing temperature and consequently more prone to futile ion cycling (Pan et al., 2008). Based on these assumptions, we suggest that bacterial cells may purposely increase their bilayer thickness in response to elevated temperatures in sediments of Spathi Bay. The rationale is that the elongation of fatty acid side chains may strengthen the relatively weak van der Waals forces in the hydrophobic region of the bilayer, thereby decreasing membrane permeability.

It is well known that microbes decrease the unsaturation levels of fatty acids with increasing temperature (Reizer et al., 1985; Suutari and Laakso, 1992; Hazel, 1995; Beranová et al., 2008). By decreasing the amount of double bonds, lipid membranes are less mobile and less fluidized, thus reducing futile ion cycling (e.g., Valentine and Valentine, 2004). This trend was indeed observed for a few lipids such as G-, PME-, and PDME-DAG (**Figure 5**). However, a number of bacterial polar lipids were observed to increase their degree of unsaturation concomitantly with an increase in fatty acid chain length. Some abundant polar lipids displaying this trend included OL and CL which represented, respectively, up to 40% and ca. 80% of total bacterial lipids in sediments of temperatures of >60°C (**Figure 3B**). This increase in unsaturation of fatty acids with increasing temperatures introduces a dilemma concerning lipid adaptation to temperature in sediments of Spathi Bay. On the one hand, an elongated chain would provide less permeability under elevated temperatures but also low membrane fluidity. On the other hand, more unsaturation implies more fluidity, but increased futile ion cycling (**Figure 6**). Alternatively, increased membrane fluidity might represent a response to increased cell curvature stress, as it is generally accepted that higher ambient temperature results in smaller individuals (e.g., Atkinson, 1994; Morán et al., 2015). Highly unsaturated fatty acids in bacterial membranes are known to localize to the curves and control its curvature stress during cell division (Kawamoto et al., 2009; Sato et al., 2012), similarly to CL (e.g., Kawai et al., 2004). Thus, curvature stress control may also explain the relatively high abundance of CL in hotter portions of the sediments (**Figure 4**), together with a general trend of increased membrane fluidity (**Figure 5**). This membrane quandary resembles the requirement for low permeability and high fluidity of archaeal membranes under heat stress discussed above. We thus suggest that archaea and bacteria may adjust both permeability and fluidity properties of their cell membranes to cope with elevated temperatures in sediments of Spathi Bay.

While monoglycosidic ceramides are minor polar lipids in the anoxic water column of the Black Sea (Schubotz et al., 2009) and in hot springs of Yellowstone National Park (Schubotz et al., 2013), phosphatidyl sphingolipids have not been reported thus far in marine sediments. We attribute the relatively high abundance of phosphate-based sphingolipids in sediments of Spathi Bay (up to 40% of total bacterial lipids; **Figures 3B**, **4B**) to their strong potential for hydrogen bonding. A pioneer study by Pascher (1976) revealed that both the amino and the hydroxyl groups of sphingolipids side chains form intermolecular hydrogen bonding with neighbor lipid molecules. More recently, nuclear magnetic resonance and MDS experiments demonstrated the additional possibility of intramolecular hydrogen bonding of amino and hydroxyl groups with the phosphatidyl headgroup of sphingomyelin (e.g., Talbott et al., 2000; Mombelli et al., 2003; Venable et al., 2014). The net effect is that sphingolipids may lend to both high internal rigidity and intermolecular order compared to DAG lipids (e.g., Pascher, 1976; Venable et al., 2014), particularly at elevated temperatures.

According to our data, the distribution of sphingolipids was markedly associated with relatively “hot” sediments (40 to 80°C, **Figures 3B**, **4B**). Indeed, sphingolipids are characteristic lipids of some thermophilic bacteria (e.g., Tenreiro et al., 1997; Yabe et al., 2013; Anders et al., 2014). Moreover, the role of sphingolipids in thermal adaptation of yeast has been demonstrated by both suppressor mutations (Wells and Lester, 1983) and their up-regulation in response to increased temperatures (Jenkins et al., 1997). Under elevated temperatures in sediments of Spathi Bay, the high melting point and hydrogen bonding potential of sphingolipids may provide a more rigid lateral organization of biological membranes when mixed with phospholipids via formation of lipid microdomains (Goñi and Alonso, 2009). Conversely, below and above the temperature threshold of 40–80°C, sphingolipids may not behave as bilayer structures when mixed with other bilayer-forming lipids (reviewed in Goñi and Alonso, 2006, 2009). Sphingolipid-mediated microdomains in bacteria (e.g., An et al., 2011; Wieland Brown et al., 2013) may thus stabilize DAG phospholipids such as CL and PC that displayed relatively high abundances at elevated temperatures (**Figures 3B**, **4B**).

### Environmental Conditions Dictate Microbial Membrane Lipid Composition: A Working Hypothesis toward a Unified Concept

Our study provides evidence for a diverse array of molecular architecture of archaeal lipids to cope with heat stress (see Polar Lipid Distribution along a Thermal Gradient in Marine Sediments). It is interesting to notice that archaea inhabiting cold to moderate temperature environments generally lack the membrane bulking features identified by our study (e.g., H-GDGT, H-nMe-GDGT; **Figure 6**). Recent advances in polar lipid analysis by HPLC-MS have allowed the description of several novel archaeal lipids (e.g., Knappy et al., 2009; Yoshinaga et al., 2011; Liu et al., 2012; Zhu et al., 2014). Those include unsaturated tetraethers, which thus far were only reported to occur in marine sediments (Zhu et al., 2014) and in a few thermophilic archaea such as Thermoplasmatales (Bauersachs et al., 2015; Yoshinaga et al., 2015b). Although the distribution of unsaturated tetraethers in the marine environment is not yet fully understood, in the studied sediments they were found more abundantly in temperatures between 30 and 55°C (**Figures 3A**, **4A**). Thus, we propose that unsaturated tetraethers do not likely reflect an archaeal membrane adaptation to extreme high temperatures, as suggested recently (Bauersachs et al., 2015). Conversely, unsaturation might be critical for motion of the rigid isoprenoidal membranes (Kellermann et al., 2016a). Supporting the idea that unsaturation of isoprenoids may not directly represent a thermal adaptation, unsaturated diethers are observed in several archaea ranging from psychrophilic to hyperthermophilic (Gonthier et al., 2001; Nishihara et al., 2002; Nichols et al., 2004; Gibson et al., 2005; Kellermann et al., 2016a).

The low permeability of archaeal membranes may not only explain the predominance of archaea in hotter sediments of Spathi Bay, but also in hotter portions of chimneys from deep-sea hydrothermal vents (Gibson et al., 2013; Reeves et al., 2014b). In those environments, a higher proportion of archaeal over bacterial lipids could be used to predict the hotter portions of chimney structures, represented in Gibson et al. (2013) by the Rainbow hydrothermal field and in Reeves et al. (2014b) by the RMR5-dark. Coincidently, both of these structures (characterized by the highest contribution of archaeal polar lipids among all samples) displayed PC as the major bacterial polar lipids, which otherwise were only found as minor components. This trend is observed in our study (**Figure 4B**), suggesting that PC may represent a common membrane lipid adaptation of bacteria to heat stress. The rationale is that temperature influences the phase behaviors of polar lipids (Quinn, 1985; Hazel and Williams, 1990): bilayer-stabilizing lamellar phase (e.g., PC, sphingolipids and 2G) or bilayer-destabilizing non-lamellar phase (e.g., PE and G). Although the acyl chain composition may also influence lipid phase behavior, increased temperatures generally induce the formation of non-lamellar phase membranes (Quinn, 1985; Hazel and Williams, 1990). Thus the ratio of membrane-stabilizing PC is predicted to increase relative to PE, and perhaps PME and PDME, with increasing growth temperatures (Hazel, 1995), as suggested by our data (**Figure 4B**).

Polar lipids of archaea in sediments of Spathi Bay were largely composed of glycolipids, likely as a thermal adaptation strategy. This glycolipid strategy for heat stress is not only consistent with cultured thermophilic archaea (Shimada et al., 2008), but also with the high abundance of archaeal glycolipids in hydrothermal systems (Gibson et al., 2013; Schubotz et al., 2013; Reeves et al., 2014b; Kellermann et al., 2016b). While the glycolipid strategy was not evidenced for bacteria in sediments of Spathi Bay, a study in Lost City Hydrothermal Field attributed the dominance of bacterial glyco- over phospholipids to a possible phosphate limitation (Bradley et al., 2009). Given that these bacteria might inhabit the mixing zone between the source fluid (pH ∼10, low phosphate and ∼90–100°C; Reeves et al., 2014a) and seawater replete with phosphate, we suggest the glycolipid strategy for thermal adaptation as an additional explanation for the dominance of bacterial glycolipids at Lost City. Given the strong hydrogen bonding network between one another, glycolipids appear as a signature lipid of many thermophilic and thermoacidophilic archaea and bacteria (e.g., Sprott et al., 1991; Yang et al., 2006; Shimada et al., 2008; Kellermann et al., 2016b). Under phosphate limitation, however, glycolipids can indeed substitute for phospholipids in photosynthetic, bacterial, and archaeal membranes (Van Mooy et al., 2006; Carini et al., 2015; Yoshinaga et al., 2015b). In addition to phosphate limitation, this glycolipid substitution has been also observed in cultured methanogens grown under hydrogen limitation (Yoshinaga et al., 2015b). We suggest that sugar headgroups may represent a common microbial strategy for energy conservation at the cell membrane level, including high temperature, low pH, phosphate limitation and perhaps substrate limitation.

Our study revealed that non-DAG lipids such as sphingolipids and OL are relatively enriched in sediments above 60°C (**Figures 3B**, **4B**). A distinctive characteristic of sphingolipids and OL compared to DAG lipids is the availability of an amino group close to the lipid headgroup in position to hydrogen bonding, which may confer an effective protection for cells under elevated temperatures (see above Discussion). Indeed, an upregulation of OL has been reported as the main membrane adaptation of cultured sulfate-reducing bacteria to increased temperatures (Seidel et al., 2013). In analogy to sphingolipids and OL, we suggest that lipid headgroups such as *N*-acetylated hexosamines and aminopentanetetrol characteristic of several deep-branching thermophilic bacteria such as *Thermus*, *Thermodesulfobacterium*, and Aquificales (e.g., Ferreira et al., 1999; Sturt et al., 2004; Yang et al., 2006) may also feature potential for strong intermolecular hydrogen bonding. This aspect could explain the widespread distribution of OL, *N*-acetylated hexosamines and aminopentanetetrol in hydrothermal settings (Gibson et al., 2013; Schubotz et al., 2013; Reeves et al., 2014b).

While ubiquitous in eukaryotic cells, sphingolipids are generally absent in most bacteria. Sphingolipid-containing bacteria, however, are highly represented in Bacteroidetes (Kato et al., 1995; Olsen and Jantzen, 2001), and sphingolipid-mediated microdomains have been linked to the capacity of *Bacteroides fragilis* to overcome stressful conditions in mammalian intestine (An et al., 2011). It is fascinating that similar properties of sphingolipids as permeability barrier in bacterial outer membranes (e.g., against antibiotics and salt; Nikaido, 2003) or as key components of “lipid rafts” in mammalian cells (e.g., Simons and Ikonen, 1997; Hannun and Obeid, 2008; Lingwood and Simons, 2010) may apply to lipid microdomains in bacterial membranes under heat stress. We suggest that sphingolipid-mediated microdomains may stabilize DAG phospholipids (e.g., CL and PC), enabling bacterial membranes to achieve both low permeability and a more fluidized configuration that are apparently required under elevated temperatures in sediments of Spathi Bay (**Figure 5**).

As described in our study, archaea and bacteria modulate their membrane architecture in response to temperature. Surprisingly, a balance between low permeability and increased fluidity (i.e., motion) appears as a unified property of microbial membranes to cope with heat stress. For instance, a higher degree of bulking (e.g., H-shaped) and fluidity (i.e., cyclization) of archaeal tetraethers were observed in concert with elevated temperatures. We propose that a more fluidized configuration of cell membranes may be beneficial for both cell bioenergetics (e.g., Valentine and Valentine, 2004, 2009; Kellermann et al., 2016a) and perhaps membrane curvature stress control (Kawamoto et al., 2009; Mouritsen, 2011; Sato et al., 2012). At the headgroup level, heat stress adaptations included membrane-stabilizing glycolipids in archaea. In bacteria, abundant CL in high temperature sediments may reflect curvature stress control and PC, OL and sphingolipids may potentially form rigid microdomains. The possibility for membrane domain formation under energy stress may provide a new dimension for interpreting lipid distribution in response to environmental conditions. That is, we are considering the possibility that lipid responses may in fact reflect a general energy conservation strategy rather than a limited effect of single stressors (e.g., temperature, pH or nutrients; see Valentine, 2007 and Kellermann et al., 2016a). It will be very exciting in future studies to interpret lipids with the idea that they are crucial for the bioenergetics of bacteria and archaea in natural systems.

## Author Contributions

MS, K-UH, and SB designed and performed research. MS, MY, SH, and RP analyzed data. All authors contributed to manuscript preparation.

## Conflict of Interest Statement

The authors declare that the research was conducted in the absence of any commercial or financial relationships that could be construed as a potential conflict of interest.

## Footnotes

1 http://www.R-project.org
