# Supplementary material for: Heat Stress Dictates Microbial Lipid Composition along a Thermal Gradient in Marine Sediments
Source: Front Microbiol. 2017 Aug 22;8:1550. doi: 10.3389/fmicb.2017.01550 (PMC5572230; doi:10.3389/fmicb.2017.01550)
Supplement: Supplementary file 1 [file Data_Sheet_1.pdf]

Supplementary Material

Heat stress dictates microbial lipid composition along a thermal gradient in marine sediments

Miriam Sollich[1]*, Marcos Y. Yoshinaga[1,2], Stefan Häusler[3], Roy E. Price[1,4], Kai-Uwe Hinrichs[1] and Solveig I. Bühring[1]

[1]University of Bremen, MARUM Center for Marine Environmental Sciences, Bremen, Germany, [2]Institute of Chemistry, University of São Paulo, São Paulo, Brazil, [3]Department of Molecular Ecology, Max Planck Institute for Marine Microbiology, Bremen, Germany, [4]School of Marine and Atmospheric Sciences, Stony Brook University, Stony Brook, NY, United States

[*]Correspondence: Miriam Sollich, msollich@uni-bremen.de

1. Supplementary Methods

1.1. Quantification of polar lipids based on response factors of standards

Microbial polar lipids were quantified by HPLC-MS and the reported concentrations corrected for response factor of commercially available or purified standards as described in S1 Table. Concentrations of mono- and diglycosidic archaeal tetraethers (including all saturated and unsaturated GDGT as well as the nMe- and/or H-(nMe)-GDGT) were corrected, respectively, for the response factor of G-GDGT 0 and 2G-GDGT 0 purified from *Archaeoglobus fulgidus* as described in Zhu et al. (2013). The monoglycosidic archaeal diether G-AR was corrected for PC-AR. Most of the bacterial polar lipids were corrected for response factors of commercial standards according to headgroup. Due to the lack of authentic standards phosphatidyl sphingolipids and ornithine lipids were corrected for the response factors of glucosylceramide and $C_{21:0}/C_{21:0}$-PC, respectively.

S1 Table. Polar lipids were quantified by HPLC-MS and concentrations were corrected for response factors based on commercial or purified standards as listed below.

#	Standard	Source	Polar Lipids*
1	G-GDGT 0	Zhu et al. (2013)	G-(H)-n(Me)-GDGT(0-Cren)
2	2G-GDGT 0	Zhu et al. (2013)	2G-(H)-n(Me)-GDGT(0-Cren)
3	PC-AR	Avanti Polar Lipids Inc., USA	G-AR
4	Glucosylceramide	Avanti Polar Lipids Inc., USA	Sphingolipids
5	DGTS	Avanti Polar Lipids Inc., USA	BL
6	$C_{16:0}/C_{16:0}$-PE	Avanti Polar Lipids Inc., USA	PE
7	$C_{16:0}/C_{16:0}$ dimethyl-PE	Avanti Polar Lipids Inc., USA	PME, PDME
8	MGlc-DAG	Avanti Polar Lipids Inc., USA	G-DAG
9	$C_{18:0}/C_{18:0}$-DGal	Biotrend Chemikalien GmbH	DGDG, SQDG
10	$C_{18:1}/C_{18:1}$-CL	Avanti Polar Lipids Inc., USA	CL
11	$C_{21:0}/C_{21:0}$-PC	Avanti Polar Lipids Inc., USA	PC, OL
12	$C_{16:0}/C_{16:0}$-PG	Avanti Polar Lipids Inc., USA	PG

#1. monoglycosyl glycerol dibiphytanyl glycerol tetraether; 2. diglycosyl glycerol dibiphytanyl glycerol tetraether; 3. 1,2-di-O-phytanyl-sn-glycero-3-phosphocholine; 4. D-glucosyl-1,1'-N-stearoyl-D-erythro-sphingosine; 5. 1,2-dipalmitoyl-sn-glycero-3-O-4'-(N,N,N-trimethyl)-homoserine; 6. 1,2-dipalmitoyl-sn-glycero-3-phosphoethanolamine; 7. 1,2-dipalmitoyl-sn-glycero-3-phosphoethanolamine-N,N-dimethyl; 8. 1,2-diacyl-3-O-(α-D-glucopyranosyl)-sn-glycerol; 9. digalactosyldiacylglycerol; 10. 1',3'-bis[1,2-dioleoyl-sn-glycero-3-phospho]-sn-glycerol; 11. 1,2-dihenarachidoyl-sn-glycero-3-phosphocholine; 12. 1,2-dipalmitoyl-sn-glycero-3-phospho-(1'-rac-glycerol). *please see Main Text for abbreviations.

1.2. Calculation of lipid indices used as input for Fig 5 (Main Text)

The percent of polar H-GDGT displayed in Fig 5 was calculated as the sum of all H-containing tetraethers (i.e. H-GDGT and H-nMe-GDGT) divided by the sum of all archaeal polar lipids. The ring index for monoglycosidic archaeal tetraethers (G-GDGT and G-H-GDGT), and respective core lipids ((H)-cGDGT) was calculated using a modified equation after Pearson et al. (2004), as follows:

$$\frac{GDGT\ 1 + 2(GDGT\ 2) + 3(GDGT0\ 3) + 4(GDGT\ 4) +\ 5(GDGT\ Cren)}{\sum(GDGT\ 0 + GDGT\ 1 + GDGT\ 2 + GDGT\ 3 + GDGT\ 4 + GDGT\ Cren)}$$

It is worth noting that crenarchaeol (Cren) was not detected among H-GDGT and thus were not used in ring index calculation for H-shaped tetraethers. A similar rationale was used to calculate the unsaturation index of monoglycosidic unsaturated GDGT:

$$\frac{GDGT\ 0{:}1 + 2(GDGT\ 0{:}2) + 3(GDGT\ 0{:}3) + 4(GDGT\ 0{:}4)}{\sum(GDGT\ 0 + GDGT\ 0{:}1 + GDGT\ 0{:}2 + GDGT\ 0{:}3 + GDGT\ 0{:}4)}$$

The methylation index or MIX was determined for G-nMe-GDGT, G-H-nMe-GDGT and H-nMe-cGDGT as follows:

$$\frac{1Me(H)GDGT + 2(2Me(H)GDGT) + 3(3Me(H)GDGT) + 4(4Me(H)GDGT)}{\sum(Me(H)GDGT + 2Me(H)GDGT + 3Me(H)GDGT + 4Me(H)GDGT)}$$

For the MIX, only the number of methylation(s) was taken into account, so that methylated tetraethers containing different number of rings were summed up.

For bacteria polar lipids, we calculated the averaged number of unsaturation(s) and chain length of lipids from specific compound classes (e.g. SQ, CL, PC). That is, the number of double bonds and chain length of each individual lipid were multiplied by the relative abundance within a compound class and summed up. Finally the diversity of polar lipids was simply estimated by the number of individual archaeal and bacterial lipids in a sample.

2. Supplementary Figures

Figure S1. *In situ* microsensor profiles of pH, oxygen and hydrogen sulfide concentrations in the upper 3 to 4 cm in sediments of Spathi Bay. For detailed information about microsensor calibration and data analysis please see Methods section. Measurements were not performed at station S2.

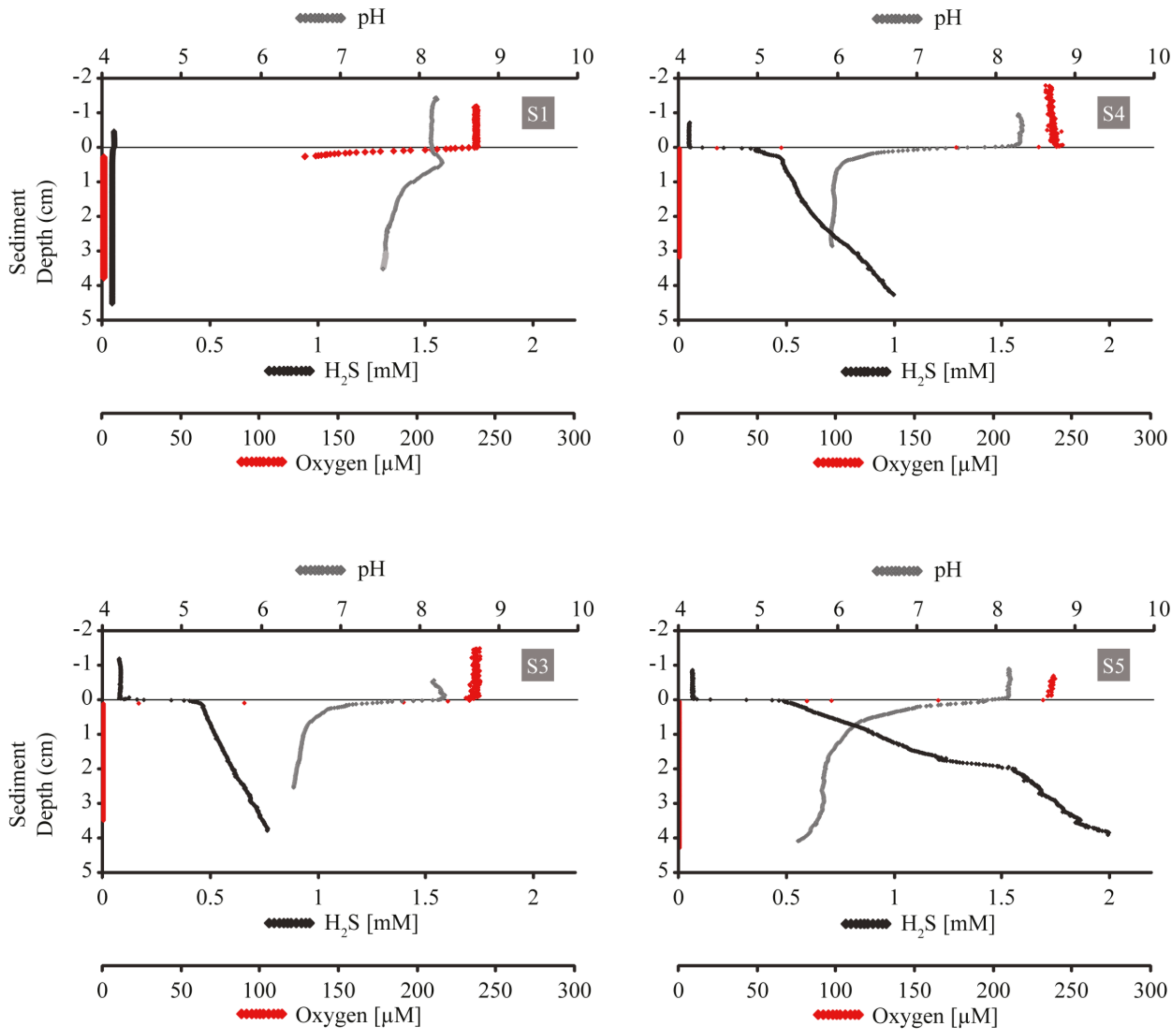

Figure S2. Non-archaeal polar lipid classes and core lipid composition of archaeal glycolipids detected in sediments of Spathi Bay. Highlighted in red are chemical structures including variable numbers of glycosidic headgroups (n), presence of unsaturation and terminal methyl branched groups in phosphatidyl sphingolipids, and tetraether modifications (unsaturation, cyclization and methylation of biphytane(s) and covalent bonds between isoprenoidal chains). List of diacylglycerolipids (DAG): (a) mono- (G) and diglycosyl (2G), n = 1 and 2, respectively; (b) sulfonoquinovosyl (SQ); (c) phosphatidylcholine (PC); (e) betaine lipids (BL); (f) phosphatidyldimethylethanolamine (PDME); (g) phosphatidylethanolamine (PE); (h) phosphatidylmethylethanolamine (PME); (i) phosphatidylglycerol (PG); (j) cardiolipin (CL). List of non-DAG lipids: (d) ornithine lipids (OL); (k) exemplary phosphatidylinositol sphingolipid (sPI). Phosphatidyl sphingolipids included PE, PG, phosphatidic acid (PA) and unknown glycosylated phosphatidyl sphingolipids (sP-Uk) as polar headgroups. List of archaeal core lipids: (l) diether or archaeaol (AR); (m) glycerol-dibiphytanyl-glycerol-tetraethers (GDGT).

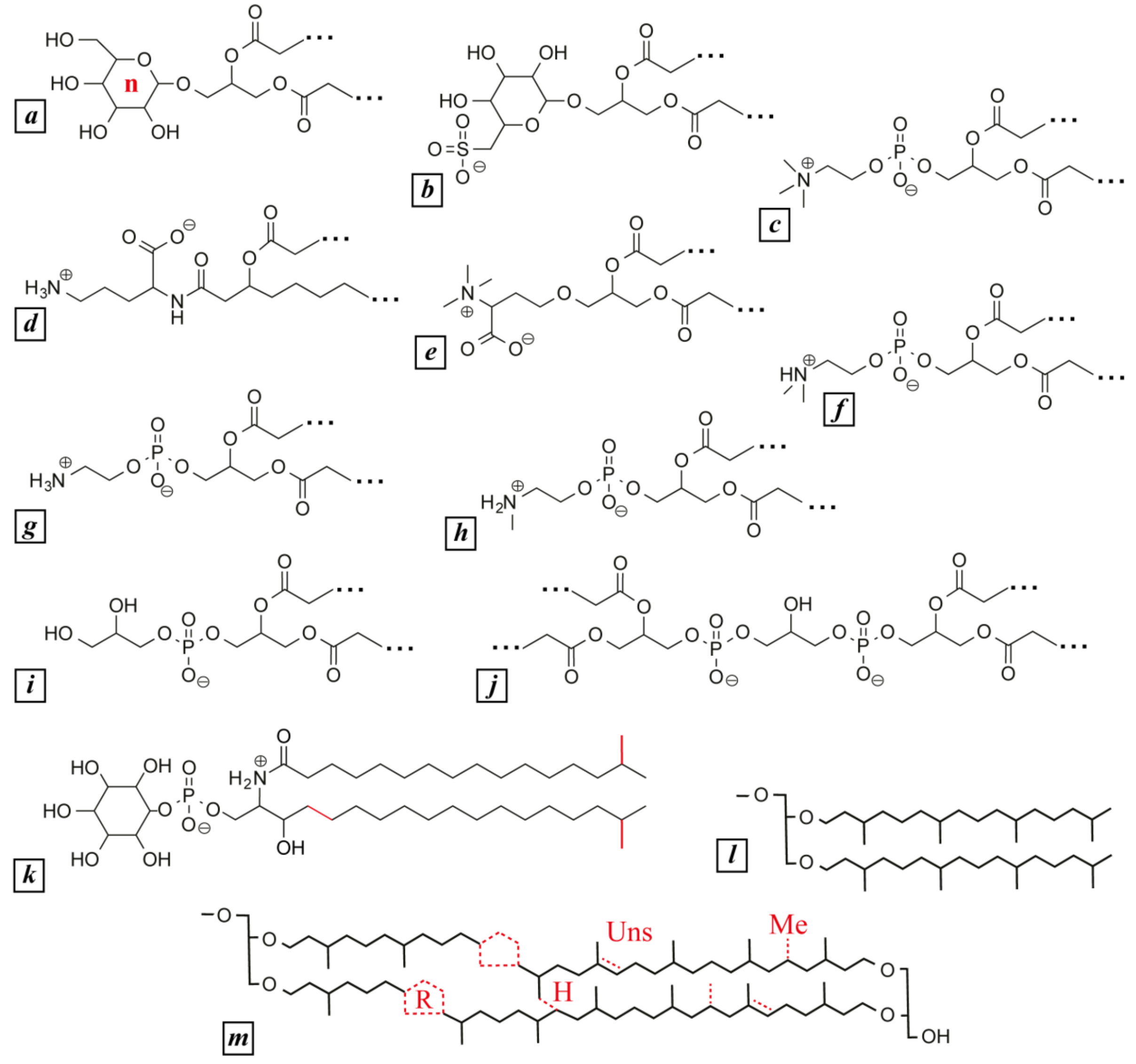

Figure S3. Fragmentation pattern of sphingomyelin from mammalian cell and phosphatidylinositol sphingolipid during MS/MS experiments in HPLC-ESI-MS. (A) MS/MS experiments of sphingomyelin typically yield fragments such as m/z 586 and 264 related respectively to ceramide (–H_2O) and sphingosine (–H_2O). (B) Tentatively identified phosphatidyl sphingolipid (sPI) structure, which was drawn according to sphingolipids of bacteria (e.g. Olsen & Jantzen, 2001; Naka et al., 2003). Note that in contrast to sphingomyelin, the MS/MS experiment of sPI yields a major ceramide fragment (m/z 536) without a water loss and no indication for a fragment related to the sphingosine as in (A) is observed. Unknown phosphatidyl sphingolipids (sP-Uk) reported in Figures 3 and 4 (Main Text) yielded MS/MS spectra with a major hexose loss from MS1, similar to those of sPI. We suggest that sP-Uk are likely similar to glycosylated phosphatidyl sphingolipids detected in *Sphingobacterium spiritivorum* (cf. Naka et al., 2003).

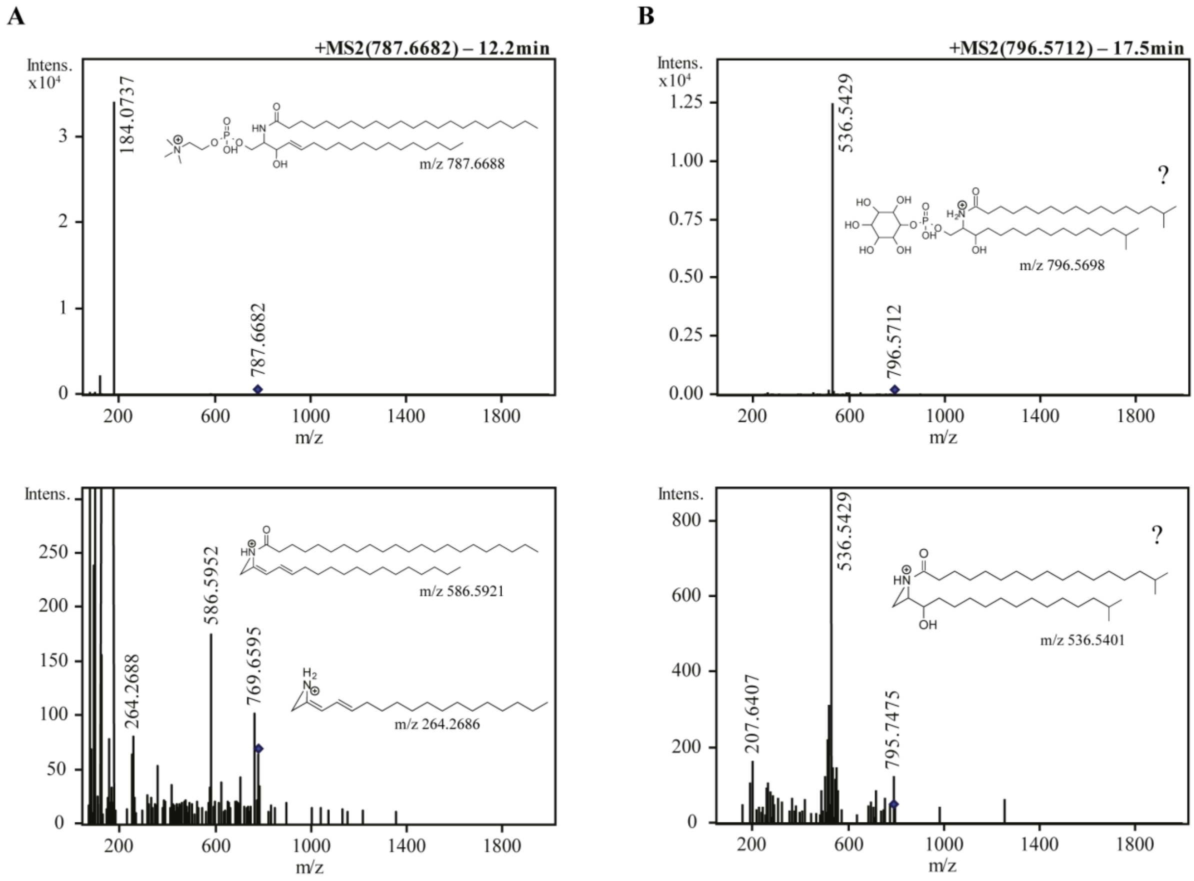

S2 Table. Spearman's rank correlation coefficient (ρ), n = number of pairs (samples) and corresponding p-value of the two-tailed tests represented in Figure 5.

n	parameter	ρ	two-sided p-value
49	% H-GDGT	0.68	2.37e-07
49	Ring H-cGDGT	0.52	1.77e-04
49	MIX H-GDGT	0.40	4.73e-03
35	Ring H-GDGT	0.35	4.27e-02
45	MIX GDGT	0.30	4.76e-02
49	Ring cGDGT	-0.68	1.96e-07
48	Uns GDGT	-0.60	6.35e-06
49	MIX H-cGDGT	-0.53	1.06e-04
48	Ring GDGT	-0.25	8.39e-02
49	Bacterial Lip Div*	-0.77	7.91e-11
49	Archaeal Lip Div*	0.60	5.86e-06
	Unsaturation:		
42	1G	-0.45	2.99e-03
36	SQ	0.34	4.32e-02
35	2G	0.76	1.17e-07
46	BL	0.25	8.93e-02
41	OL	0.70	1.15e-06
44	PE	0.66	8.92e-07
33	PME	-0.52	2.27e-03
26	PDME	-0.63	5.22e-04
47	PC	-0.03	8.24e-01
38	PG	0.33	4.28e-02
37	CL	0.49	2.03e-03
30	sPG	0.26	1.71e-01
30	sPE	0.76	2.68e-06
12	sPA	0.50	9.88e-02
24	sPI	0.42	4.23e-02
	Chain length:		
42	1G	0.03	8.30e-01
36	SQ	0.54	5.91e-04
35	2G	0.74	4.32e-07
46	BL	0.11	4.61e-01
41	OL	0.72	4.95e-07
44	PE	0.53	2.16e-04
33	PME	0.05	8.03e-01
26	PDME	0.66	3.32e-04
47	PC	0.43	2.51e-03
38	PG	0.42	9.42e-03

37	CL	0.56	3.08e-04
30	sPG	0.42	1.98e-02
30	sPE	0.96	2.20e-16
12	sPA	0.49	1.10e-01
24	sPI	0.91	2.61e-06

* Lip Div = Lipid Diversity

3. References

Naka, T., Fujiwara, N., Yano, I., Maeda, S., Doe, M., Minamino, M., et al. (2003). Structural analysis of sphingophospholipids derived from *Sphingobacterium spiritivorum*, the type species of genus *Sphingobacterium*. *Biochim. Biophys. Acta* 1635, 83-92. doi: 10.1016/j.bbalip.2003.10.010

Olsen, I., and Jantzen, E. (2001). Sphingolipids in bacteria and fungi. *Anaerobe* 7, 103-112. doi: 10.1006/anae.2001.0376

Pearson, A., Huang, Z., Ingalls, A. E., Romanek, C. S., Wiegel, J., Freeman, K. H., et al. (2004). Nonmarine crenarchaeol in Nevada hot springs. *Appl. Environ. Microbiol.* 70, 5229-5237. doi: 10.1128/AEM.70.9.5229-5237.2004

Zhu, C., Lipp, J. S., Wörmer, L., Becker, K. W., Schröder, J., and Hinrichs, K.-U. (2013). Comprehensive glycerol ether lipid fingerprints through a novel reversed phase liquid chromatography-massspectrometry protocol. *Org. Geochem.* 65, 53-62. doi:10.1016/j.orggeochem.2013.09.012
